# Genetic Basis of Hidden Phenotypic Variation Revealed by Increased Translational Readthrough in Yeast

**DOI:** 10.1371/journal.pgen.1002546

**Published:** 2012-03-01

**Authors:** Noorossadat Torabi, Leonid Kruglyak

**Affiliations:** 1 Lewis-Sigler Institute for Integrative Genomics, Princeton University, Princeton, New Jersey, United States of America; 2 Department of Molecular Biology, Princeton University, Princeton, New Jersey, United States of America; 3 Department of Ecology and Evolutionary Biology, Princeton University, Princeton, New Jersey, United States of America; 4 Howard Hughes Medical Institute, Princeton University, Princeton, New Jersey, United States of America; University of California San Francisco and Howard Hughes Medical Institute, United States of America

## Abstract

Eukaryotic release factors 1 and 3, encoded by *SUP45* and *SUP35*, respectively, in *Saccharomyces cerevisiae*, are required for translation termination. Recent studies have shown that, besides these two key factors, several genetic and epigenetic mechanisms modulate the efficiency of translation termination. These mechanisms, through modifying translation termination fidelity, were shown to affect various cellular processes, such as mRNA degradation, and in some cases could confer a beneficial phenotype to the cell. The most studied example of such a mechanism is [PSI^+^], the prion conformation of Sup35p, which can have pleiotropic effects on growth that vary among different yeast strains. However, genetic loci underlying such readthrough-dependent, background-specific phenotypes have yet to be identified. Here, we used *sup35^C653R^*, a partial loss-of-function allele of the *SUP35* previously shown to increase readthrough of stop codons and recapitulate some [PSI^+^]-dependent phenotypes, to study the genetic basis of phenotypes revealed by increased translational readthrough in two divergent yeast strains: BY4724 (a laboratory strain) and RM11_1a (a wine strain). We first identified growth conditions in which increased readthrough of stop codons by *sup35^C653R^* resulted in different growth responses between these two strains. We then used a recently developed linkage mapping technique, extreme QTL mapping (X-QTL), to identify readthrough-dependent loci for the observed growth differences. We further showed that variation in *SKY1*, an SR protein kinase, underlies a readthrough-dependent locus observed for growth on diamide and hydrogen peroxide. We found that the allelic state of *SKY1* interacts with readthrough level and the genetic background to determine growth rate in these two conditions.

## Author Summary

Proper termination is an important step in a successful mRNA translation event. Many factors, employing genetic and epigenetic mechanisms, are involved in modifying translation termination efficiency in the budding yeast, *Saccharomyces cerevisiae*. [PSI^+^], the prion conformation of Sup35p, one of the translation termination factors in yeast, provides an example of such mechanisms. [PSI^+^] increases readthrough of stop codons. This has the potential to unveil hidden genetic variation that may enhance growth in some yeast strains in certain environments. The specific details of readthrough-dependent phenotypes, however, have remained poorly understood. Here, we used a partial loss-of-function allele of *SUP35*, which increases readthrough of stop codons, and a recently developed linkage mapping technique, X-QTL, to map loci underlying readthrough-dependent growth phenotypes in two divergent yeast strains, BY (a laboratory strain) and RM (a wine strain). We found that readthrough-dependent growth phenotypes are often complex, with multiple loci influencing growth. We also showed that variants in the gene *SKY1* underlie one of the loci detected for readthrough-dependent growth phenotypes in the presence of two chemicals that induce oxidative stress.

## Introduction

High fidelity in translation, one of the key steps in the expression of genetic information, is essential for functional integrity of the cell. Efficient termination is an important aspect of translational fidelity, and a multitude of factors participate in this process [1], [2]. The efficiency of translation termination depends on the competition between stop codon recognition by release factors and decoding by near-cognate tRNAs (tRNAs that can pair with two of the three bases of the stop codon) [3]. Recent studies of translation termination in *Saccharomyces cerevisiae* have revealed genetic and epigenetic regulatory mechanisms that modify translation termination efficiency, which can affect cellular processes such as mRNA degradation and, in some cases, can confer a beneficial phenotype to the cell [4]. The most studied example of such mechanisms is the yeast prion [PSI^+^], which is formed by a conformational change in Sup35p, a subunit of the translation termination complex [5].

[PSI^+^] is an epigenetic modifier of translation termination efficiency in *S. cerevisiae* [6]. Sup35p carries an intrinsically disordered prion-determining region at its amino terminus. When this domain switches to the aggregating amyloid conformation (the prion conformation), much of the protein becomes unavailable for translation terminations, which in turn increases readthrough of stop codons [7], [8]. [PSI^+^] was reported to generate different phenotypes in different genetic backgrounds, and most of these phenotypic effects were shown to be recapitulated by a partial loss-of-function allele of *SUP35*, *sup35^C653R^* [9].

Previous studies have shown that some of the observed [PSI^+^]-dependent phenotypic effects are due to ribosomal frame-shifting [10]. It has also been proposed that some of the observed phenotypic variation in different yeast strains can be due to [PSI^+^]-dependent increase in readthrough, which results in ribosomes bypassing stop codons and reading into regions such as sequences at the 3′ untranslated regions or pseudogenes [11]. These regions are thought to be under less selective pressure than coding sequences, and therefore may be more divergent among different yeast strains. However, specific loci underlying phenotypic differences due to increased readthrough of stop codons have yet to be identified.

Here, we used *sup35^C653R^* to examine the phenotypic effects of decreasing translation termination efficiency in various growth conditions in two divergent yeast strains, BY4724 (a laboratory strain hereafter referred to as BY) and RM11_1a (a wine strain hereafter referred to as RM). Using a quantitative dual luciferase assay [12], we showed that this partial loss-of-function allele of *SUP35* increased readthrough of stop codons, as previously reported [13]. We identified nine growth conditions (about one quarter of the growth conditions tested) in which increased readthrough of stop codons resulted in different growth responses between BY and RM. Then, we used a recently developed linkage mapping technique, extreme QTL mapping (X-QTL) [14], to find the genetic basis for the observed readthrough-dependent growth differences. We found one to six readthrough-dependent loci for the growth conditions examined, suggesting that phenotypes revealed by increased translational readthrough are often genetically complex. We further showed that variation in *SKY1* underlies a readthrough-dependent locus observed for growth in diamide and hydrogen peroxide. We found that a complex interplay between *sup35*-mediated increase in readthrough, the allelic state of *SKY1*, and genetic background determines growth in these two conditions. Our results provide new insights into the genetic basis of phenotypes revealed by decreased translation termination efficiency in yeast.

## Results

### Effect of increased readthrough on growth in some conditions varies with genetic background

To compare the growth effects of increased readthrough in BY and RM, we replaced the wildtype allele of *SUP35* in each strain with a partial loss-of-function allele, *sup35^C653R^*. Sequence comparison showed no differences between the BY and RM amino acid sequences of *SUP35* in either the N-terminal (prion forming) domain or the C-terminal (translation termination) domain. We previously showed that the baseline readthrough level is different between BY and RM [15]. We used a quantitative dual luciferase assay [12] to show that *sup35^C653R^* increases readthrough in both strains by approximately four folds (Figure S1). We then measured growth rates of BY and RM carrying the wildtype alleles of *SUP35* (hereafter “wildtype”) and *sup35^C653R^* (hereafter “*sup35*”). We tested 33 different growth conditions including alternative carbon sources, different temperatures, and growth in the presence of small molecules that perturb varied cellular processes (Table S1). These growth conditions have previously been shown to induce different growth phenotypes in isogenic [PSI^+^] and [psi^−^] strains [16] and/or different growth phenotypes in BY and RM [17]. For each genetic background, we then calculated the ratio of the *sup35* strain growth rate and the wildtype strain growth rate (hereafter, “growth rate ratio”).

**Figure 1:**
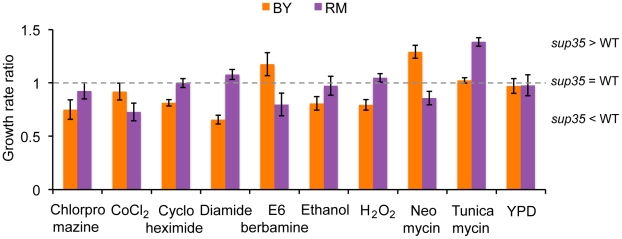
Readthrough-dependent strain-specific growth effects. The ratio of *sup35* strain growth rate and wildtype strain growth rate is plotted (mean ± SD) for BY (orange bars) and RM (purple bars) for the nine conditions in which the effect of *sup35^C653R^*-mediated increase in readthrough on growth differed between the two strain backgrounds (uncorrected *p*<0.05, FDR∼10%).

We found that in control medium (YPD), the growth rate ratios for both strains were not significantly different from one (Figure 1). This showed that in both strains, *sup35*-mediated increase in readthrough had no significant effect on growth rates in rich medium. For 24 out of 33 growth conditions tested, we found that the *sup35*-mediated increase in readthrough had the same effect on growth rate ratio in BY and RM; it decreased or did not alter either strain's growth rate ratio (data not shown). However, we found nine growth conditions in which the growth rate ratio was significantly different between BY and RM (uncorrected *p*<0.05; False Discovery Rate (FDR) ∼10%; Figure 1). In five growth conditions (chlorpromazine, cobalt chloride, cycloheximide, ethanol and hydrogen peroxide) increase in readthrough did not alter growth rate in one strain (growth rate ratio not significantly different from one) while it decreased growth rate in the other strain (growth rate ratio significantly less than one). In the presence of tunicamycin, increase in readthrough did not change growth rate in BY (growth rate ratio not significantly different from one) while it increased growth rate in RM (growth rate ratio significantly greater than one). In the remaining three growth conditions (diamide, E6-berbamine, and neomycin), increase in readthrough increased growth rate in one genetic background while decreasing growth rate in the other (Figure 1).

### X-QTL reveals readthrough-dependent loci

We used X-QTL [14] to examine the genetic basis of the observed readthrough-dependent differences in growth rate ratio between BY and RM. For each growth condition, we performed X-QTL on two segregant pools in parallel: a wildtype pool from a cross between wildtype BY and RM, and a *sup35* pool from a cross between BY and RM both carrying *sup35^C653R^* (Materials and Methods). We grew these pools on selection plates (rich medium plus the chemical agent of interest) and control plates (Materials and Methods), and compared the allele frequencies between the selected pools and control pools by microarray-based single nucleotide polymorphism (SNP) genotyping as previously described [14]. A locus that affects growth rate in a given condition independent of *sup35* is expected to be detected as an allele frequency skew of similar direction and magnitude in both the wildtype and *sup35* selected pools. In contrast, a locus whose effects depend on *sup35* is expected to show a difference in the allele frequency skew between the two pools.

**Figure 2:**
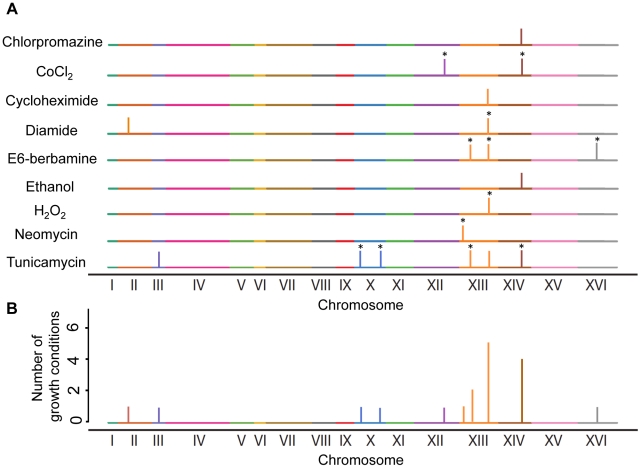
Readthrough-dependent loci. A) Loci detected for each growth condition as significantly different between X-QTL allele frequencies in wildtype and *sup35* selected pools (FDR 5%, Materials and Methods). Readthrough-dependent loci that were also called significant by an alternative statistical approach are marked with *. B) Histogram of the number of growth conditions for each readthrough-dependent locus.

The number of loci detected for growth rates in the nine conditions at an FDR of 5% ranged from one to 20 in both wildtype and *sup35* crosses (Figure S2A–S2I). The results in the wildtype cross were similar to those previously described for these growth conditions [14], which showed the reproducibility of X-QTL. Most loci showed similar allele frequency skews in the wildtype and *sup35* pools; however, 18 loci showed significant differences between these pools (FDR = 5%, Materials and Methods) (Figure S2A–S2I). We refer to these loci as “readthrough-dependent”. One to six readthrough-dependent loci were detected in the growth conditions tested (Figure 2A). These results showed that *sup35*-mediated effects on growth in certain conditions are genetically complex, as was previously suggested for some [PSI^+^]-dependent growth phenotypes [9]. Each readthrough-dependent locus had an effect in one to five conditions, with a total of ten distinct loci detected (Figure 2B).

### *SKY1* variation underlies the readthrough-dependent locus on chromosome XIII

In order to gain more insight into readthrough-dependent effects on growth rate, we focused on the locus on the right arm of chromosome XIII, which affected growth in five conditions (Figure 2B). We chose one of the growth conditions in which this locus had the strongest effect for further investigation: diamide, a sulfhydryl-oxidizing agent [18] (Figure S2D). At this locus, we detected a frequency skew in favor of the RM allele in the *sup35* pool but not in the wildtype pool, which suggested that in the presence of increased readthrough, strains carrying an RM allele at this locus grow better on diamide than strains carrying the BY allele.

Based on sequence comparison between BY and RM for the genes in this region (Figure S3), we selected *SKY1* and *MRE11* for further investigation, as both contained nonsynonymous changes in their open reading frames and in the downstream regions that might be translated due to increased readthrough. Comparison of the coding sequence of *SKY1* between BY and RM showed 13 single nucleotide polymorphisms (SNPs) between the two strains, including seven nonsynonymous substitutions. The downstream sequence contained a two-nucleotide deletion in BY at the 129^th^ nucleotide after stop codon, which results in the addition of three amino acids in BY before the next stop codon is reached. Comparison of the coding sequence of *MRE11* between BY and RM showed eight SNPs between the two strains, including five nonsynonymous substitutions. The downstream sequence contained five SNPs, including four nonsynonymous substitutions.

**Figure 3:**
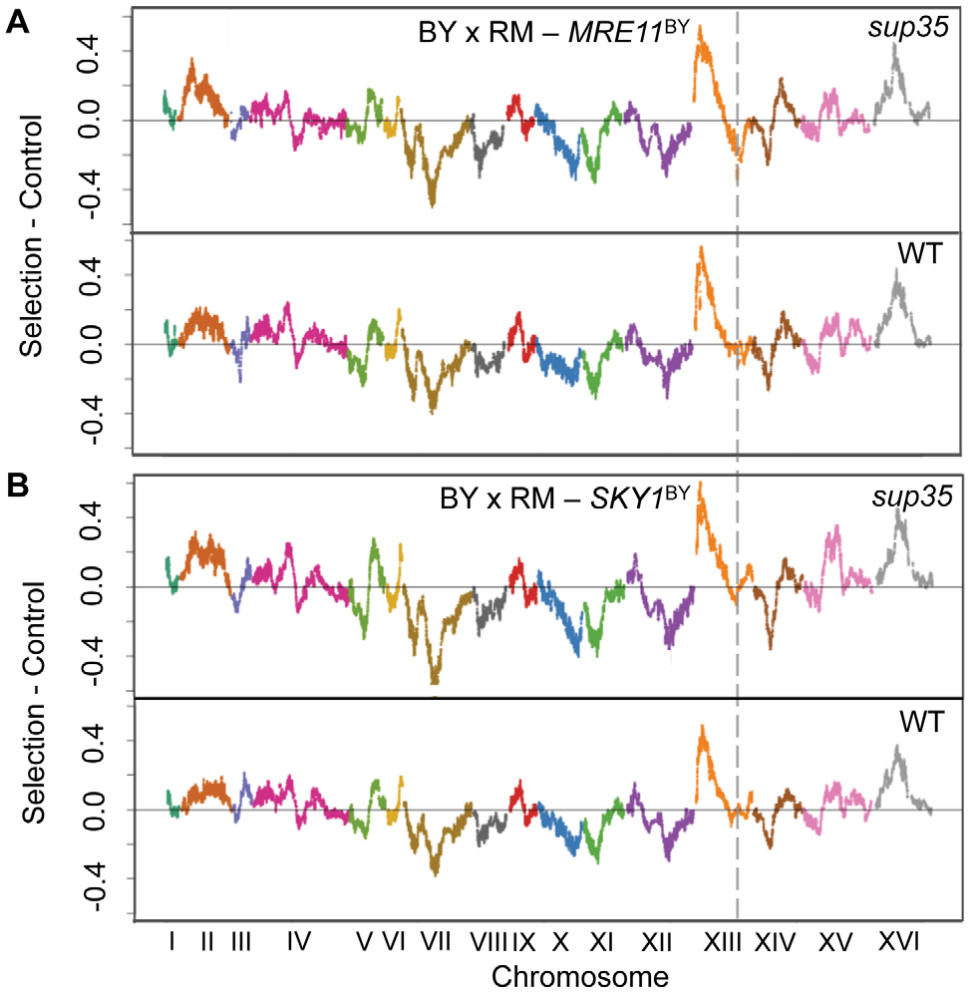
*SKY1* variation underlies the locus on Chromosome XIII for growth on diamide. A) Comparisons of the selected segregating population (Selection) to the whole population (Control) from a cross between *sup35* BY and RM parent strains (*sup35*) and a cross between wildtype parent strains (WT) are shown for *MRE11*-fixed populations (BY×RM- *MRE11* ^BY^) in presence of diamide. For plotting, average of two biological replicates is used for each selection and control. Sliding window averages (40 kb) are plotted. Enrichment of the BY allele is indicated by deviations above zero and enrichment of the RM allele is indicated by deviations below zero. B) Comparisons of the selected segregating population (Selection) to the whole population (Control) from a cross between *sup35* BY and RM parent strains (*sup35*) and a cross between wildtype parent strains (WT) are shown for *SKY1*-fixed populations (BY×RM- *SKY1* ^BY^) in presence of diamide. Average of two biological replicates is used for each selection and control. The dotted line shows the interval surrounding *MRE11* and *SKY1*.

To test the causality of *SKY1* and *MRE11* polymorphisms for the effects of this locus, we replaced the *SKY1* and *MRE11* genes in both wildtype and *sup35* RM with the BY versions. For both genes, we replaced the downstream sequence along with the coding sequence. We previously showed that the expression level of *SKY1* is lower in RM than in BY, and that this difference maps to the location of the *SKY1* gene, suggesting the presence of a *cis*-regulatory polymorphism [19]. Therefore, we included the upstream regulatory sequence in the *SKY1* allele replacement along with the coding and downstream sequence. We then repeated the X-QTL experiments for growth on diamide with both wildtype and *sup35* segregant pools from crosses using the RM parent strains with *SKY1* and *MRE11* allele replacements (that is, both parents carried the BY allele of *SKY1* or *MRE11*, respectively). In the crosses with the *MRE11* allele replacement, the results were unchanged; that is, we still saw a skew in the direction of the RM allele at this locus in the *sup35* pool despite the fact that *MRE11* was no longer polymorphic, ruling it out as the causal gene for this locus (Figure 3A). In contrast, this allele frequency skew disappeared in the *sup35* pool from the cross with the *SKY1* allele replacement, and there was no longer any difference in allele frequency at this locus between the wildtype and *sup35* pools (Figure 3B). These results demonstrate that polymorphisms in *SKY1* are causal for the effects of this locus, and that the difference in growth between the RM and BY alleles of *SKY1* is revealed when readthrough is increased from the wildtype level by *sup35^C653R^*.

**Figure 4:**
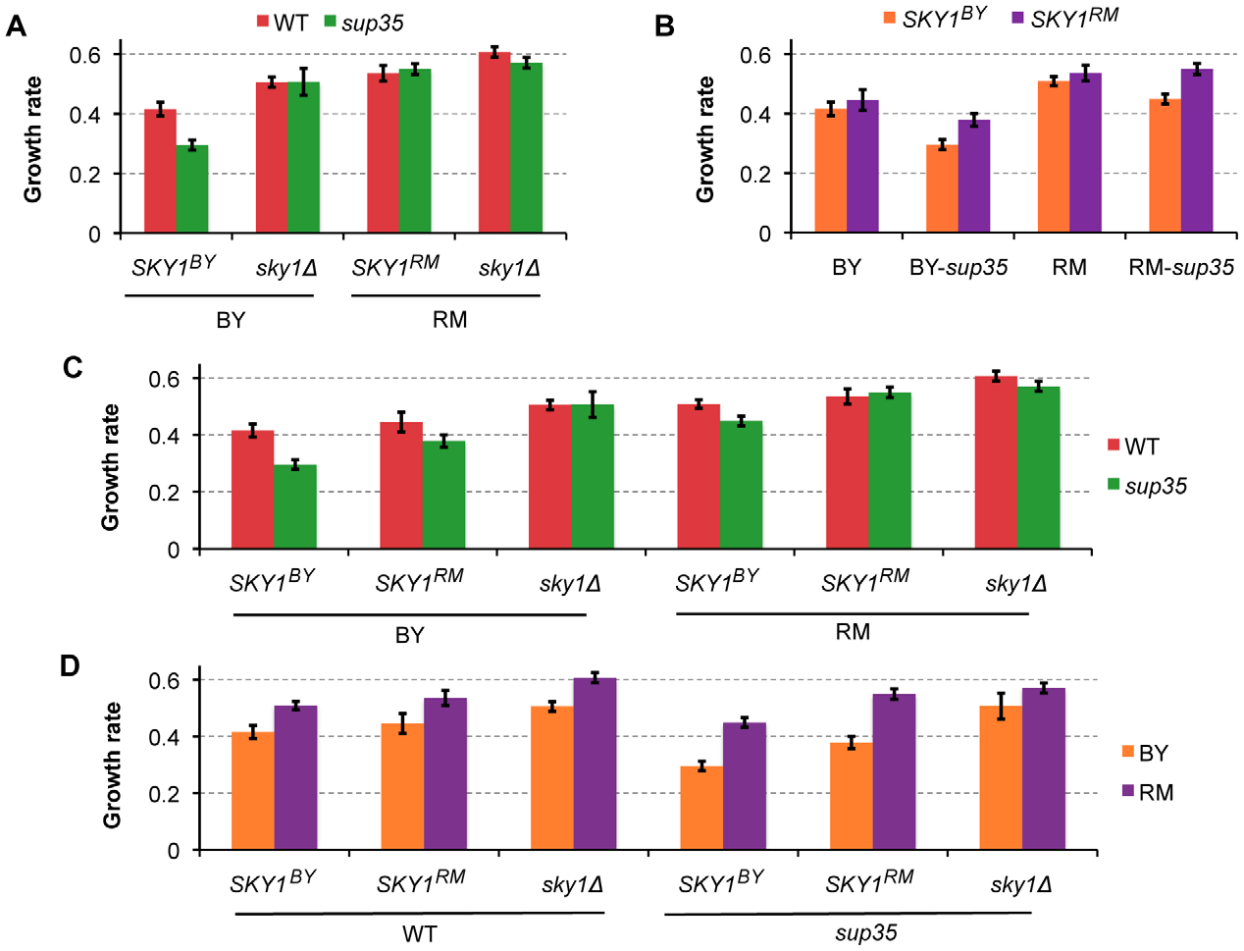
*SKY1* variation contributes to readthrough-dependent differences in growth rates of BY and RM on diamide. A) Knocking out *SKY1* eliminates the observed difference between wildtype and *sup35* growth rates in BY background. B) Replacing *SKY1* (upstream regulatory region, the open reading frame and the downstream sequence) in the BY background with the RM allele increased growth rate in diamide in the presence of *sup35*, and replacing *SKY1* (upstream regulatory region, the open reading frame and the downstream sequence) in the RM background with the BY allele decreased growth rate in the presence of *sup35*. C and D) these panels show the data presented in panels A and B grouped in different ways to highlight the growth effects of the *sup35* allelic state and the growth effects of the genetic background. Growth rates of wildtype and *sup35* BY and RM, as well as the corresponding *SKY1* swapped and *sky1Δ* strains are shown for growth in the presence of diamide, grouped according to the *sup35* allelic state (C) and genetic background (D). For each strain, growth rates are normalized based on the strain's growth rate in rich medium (YPD).

To better understand the effect of *SKY1* on the readthrough-dependent difference in growth rate between BY and RM on diamide, we measured growth rates of *SKY1*-swapped wildtype and *sup35* BY and RM and compared them to the original strains. For this experiment, we constructed wildtype and *sup35* BY strains carrying the RM allele of *SKY1*. Similar to the previous replacements strains, we replaced the upstream regulatory region along with the coding and downstream sequences of *SKY1.* We also measured growth rates of wildtype and *sup35* BY and RM strains with *SKY1* knocked out (*sky1*Δ strains). In the presence of *sky1*Δ, there was no significant difference in growth between wildtype and *sup35* strains in either genetic background (Figure 4A) implying that readthrough-dependent differences in growth are mediated by *SKY1*. As expected from the X-QTL results, replacing *SKY1* in the BY background with the RM allele increased growth rate in diamide in the presence of *sup35*, and replacing *SKY1* in the RM background with the BY allele decreased growth rate in the presence of *sup35* (Figure 4B). These results confirmed the growth effects of *SKY1* polymorphisms.

A readthrough-dependent locus for growth on hydrogen peroxide was also observed at this genomic location (Figure 2B, Figure S2G). Therefore, we tested whether *SKY1* polymorphisms underlie the effects of this locus on hydrogen peroxide as well. X-QTL experiments with allele replacement strains showed that polymorphisms in *SKY1* are indeed causal (Figure S4). Growth rate measurements in wildtype and *sup35* BY and RM strains and the corresponding *SKY1* swapped and *sky1*Δ strains confirmed the effects of *SKY1* polymorphisms on growth in the presence of hydrogen peroxide (Figure S5A–S5B).

### Complex interplay between *sup35* effects, allelic state of *SKY1*, and genetic background determines growth rate on diamide and hydrogen peroxide

When we first measured the growth rates of wildtype and *sup35* BY and RM in the presence of diamide, we found that BY-*sup35* grew significantly slower than wildtype BY, while there was little difference in growth rate between RM-*sup35* and wildtype RM (Figure 4C). When the BY allele of *SKY1* was replaced with the RM allele, the difference in growth between the *sup35* and wildtype strains was reduced, although it remained significant (Figure 4C). When the RM allele of *SKY1* was replaced with the BY allele, the *sup35* strain grew somewhat slower than the wildtype strain (Figure 4C). We found similar results for growth of these strains in presence of hydrogen peroxide (Figure S5C). The observation that *SKY1* replacement strains recapitulated the direction but not the magnitude of the effects of readthrough on growth in these conditions seen in the parent strains suggested the presence of interactions between *SKY1* polymorphisms, readthrough, and genetic background. We used Analysis of Variance (ANOVA) (Materials and Methods) to formally test the effects of these factors and the interactions among them (Table 1, Table S2). The model showed a major effect of the genetic background, with RM growing better in presence of diamide and hydrogen peroxide than BY. *SKY1* allelic status also had a significant effect, with the RM allele increasing growth. Readthrough level did not show a significant effect on its own but did show significant interaction effects with both genetic background and *SKY1* allelic status.

**Table 1:** Modeling growth rate in the presence of diamide using ANOVA with three factors: genetic background (BG), *SUP35* allelic status, and *SKY1* allelic status.

Coefficients	Estimate	Std. Error	t value	Pr (>|t|)
(Intercept)	0.550	0.00796	69.11	<2e-16
BG (BY)	−0.171	0.0113	−15.2	<2e-16
SUP35 (WT)	−0.0141	0.0113	−1.25	0.216
SKY1 (BY)	−0.101	0.0113	−8.95	2.20E-12
BG (BY): SUP35 (WT)	0.0808	0.0159	5.08	4.55E-06
BG (BY): SKY1 (BY)	0.0172	0.0159	1.08	0.283
SUP35 (WT): SKY1 (BY)	0.0739	0.0159	4.64	2.12E-05
BG (BY): SUP35 (WT): SKY1 (BY)	−0.0203	0.0225	−0.903	0.371

These results suggest a complex interplay between the effects of *sup35*-mediated increase in readthrough, the allelic state of *SKY1*, and genetic background in determining growth on diamide and hydrogen peroxide. At wildtype readthrough levels, RM grows better than BY, and swapping *SKY1* in either strain with the version from the other strain has little effect (Figure 4D, Figure S5D). When readthrough is increased by introduction of *sup35^C653R^*, growth rate decreases dramatically in BY, but shows no change in RM (Figure 4D, Figure S5D). The slower growth rate of BY-*sup35* in comparison to wildtype BY is partially rescued by introduction of the RM allele of *SKY1* and completely rescued by knocking out *SKY1* (Figure 4D, Figure S5D). Introduction of the BY allele of *SKY1* into RM-*sup35* reduces the growth rate of this strain but not to the extent seen in the BY background (Figure 4D, Figure S5D). Thus, *sup35^C653R^* and the BY allele of *SKY1* act together to lower growth rate on diamide and hydrogen peroxide, and this effect is accentuated by as yet unidentified factors in the BY genetic background.

**Figure 5:**
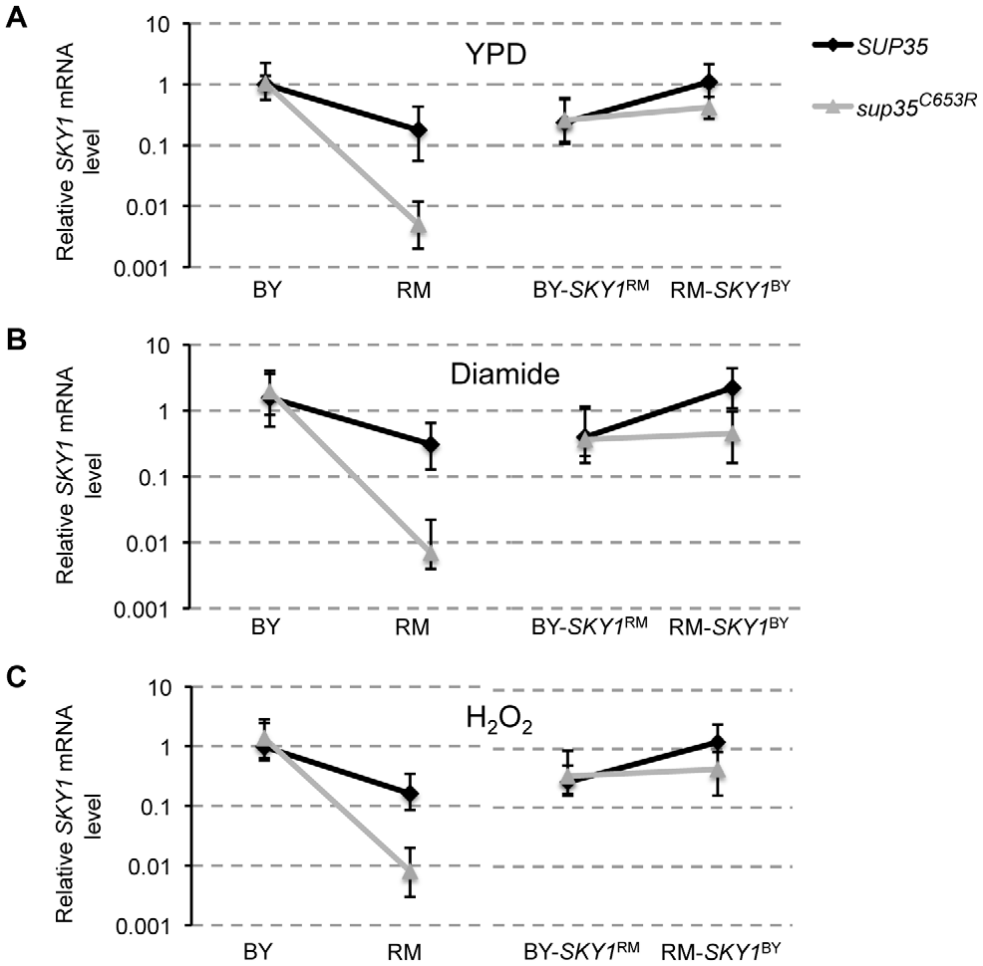
Measured *SKY1* mRNA levels in YPD, diamide, and hydrogen peroxide. *SKY1* mRNA level measured via quantitative RT-PCR is compared for wildtype (WT) and *sup35* BY and RM as well as BY-*SKY1* ^RM^ and RM-*SKY1* ^BY^ in rich media (A), rich media plus diamide (B) and rich media plus hydrogen peroxide (C). Measurements are shown relative to the mRNA levels in wildtype BY in YPD. *SKY1* replacement strains were made by swapping the *SKY1* upstream regulatory region, open reading frame, and downstream sequence.

Given the evidence for *cis*-regulatory polymorphism in *SKY1* that lowers expression of the RM allele, we investigated whether differences in transcript abundance could account for the allelic effect of *SKY1*. We used quantitative RT-PCR to measure *SKY1* mRNA levels in wildtype and *sup35* BY and RM, as well as in the corresponding *SKY1*-replaced strains (Materials and Methods). We found that *SKY1* mRNA levels were independent of the growth condition used (Figure 5). As expected based on microarray data [19] we found that *SKY1* expression is higher in wildtype BY than in wildtype RM. Moreover, we found that swapping *SKY1* in wildtype BY and wildtype RM with the alternate allele changed *SKY1* expression level to the alternative level. These results confirm the presence of *cis*-regulatory polymorphism that alters the expression level of *SKY1* between BY and RM. Surprisingly, in the RM background, increasing readthrough from the wildtype level to the *sup35* level resulted in roughly a ten-fold drop in *SKY1* expression level, while no change was observed in BY-*sup35*. This drop in *SKY1* mRNA in RM-*sup35* was largely reversed by swapping in the BY allele of *SKY1* (Figure 5). We used ANOVA to model the effect of the measured *SKY1* mRNA abundance on growth rate, and then used the residual growth rate to test whether the allelic effect of *SKY1* was changed. The results suggested that the readthrough-dependent growth effects of *SKY1* are not mediated by mRNA levels (Table 2, Table S3).

**Table 2:** Modeling growth rate in the presence of diamide after regressing out *SKY1* mRNA data using ANOVA with three factors: genetic background (BG), *SUP35* allelic status, and *SKY1* allelic status.

Coefficients	Estimate	Std. Error	t value	Pr (>|t|)
(Intercept)	0.0802	0.00973	8.19	3.71E-11
BG (BY)	−0.160	0.0138	−11.57	<2e-16
SUP35 (WT)	−0.00604	0.0138	−0.436	0.665
SKY1 (BY)	−0.0868	0.0138	−6.27	5.50E-08
BG (BY): SUP35 (WT)	0.0737	0.0196	3.76	4.10E-04
BG (BY): SKY1 (BY)	0.0299	0.0196	1.53	0.133
SUP35 (WT): SKY1 (BY)	0.101	0.0196	5.14	3.67E-06
BG (BY): SUP35 (WT): SKY1 (BY)	−0.038	0.0277	−1.37	0.176

**Figure 6:**
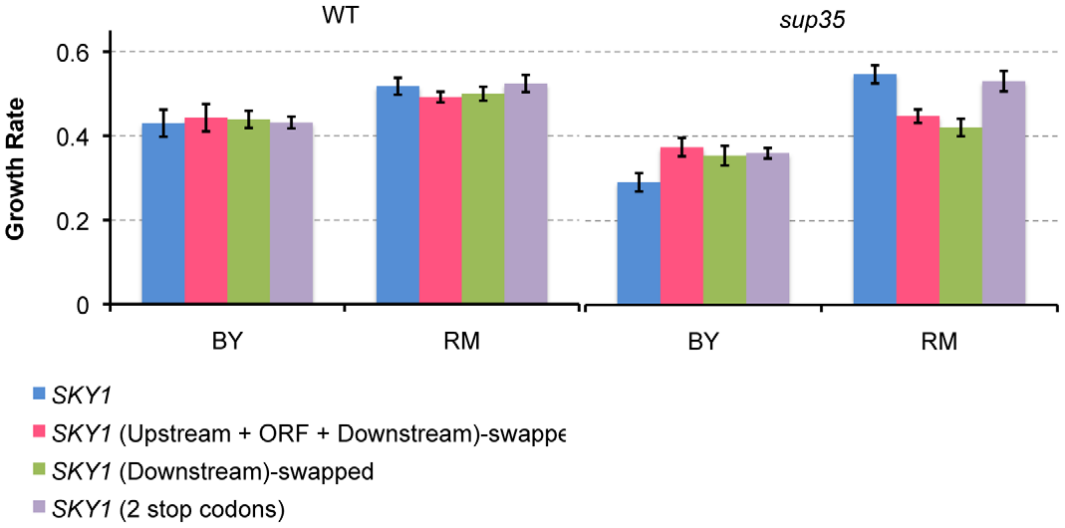
Readthrough-dependent strain-specific growth effects of *SKY1* in diamide. Replacing the *SKY1* downstream sequence alone captures the allelic effects of *SKY1* in *sup35* strains. Introducing a second stop codon immediately after the native stop codon at the end of the *SKY1* open reading frame shows that strain-specific growth effects of *SKY1* polymorphism are readthrough-dependent.

To gain further mechanistic insight into how *sup35^C653R^* leads to the differential allelic effects of *SKY1*, we swapped just the downstream sequences of *SKY1* in both wildtype and *sup35* BY and RM with the alternative alleles. These replacement strains differ from the parent strains only by the polymorphism that extends the C-terminus. Swapping this polymorphism alone captured the allelic effect of *SKY1* in both growth conditions (Figure 6, Figure S6), consistent with a differential effect of readthrough on the downstream regions from the two strains. To test whether the allelic effect of *SKY1* is directly related to translational readthrough, we then introduced a second stop codon immediately after the native stop codon in wildtype and *sup35* BY and RM strains. Introducing a second stop codon in wildtype BY and RM did not affect growth rates of these strains in diamide or hydrogen peroxide (Figure 6, Figure S6). In the presence of the *sup35* mutation, the growth rate in the BY strain with the second stop codon rose to the same level as when the *SKY1* allele is replaced with the RM version, while the second stop codon did not alter growth rate in RM (Figure 6, Figure S6). These results support the hypothesis that increased readthrough of the BY downstream region due to the *sup35* mutation causes reduced growth in diamide and hydrogen peroxide, perhaps because translation of this region stabilizes Sky1p.

## Discussion

Modifying translational readthrough in *S. cerevisiae* has been shown to affect yeast cells in various ways [4]. The prion [PSI^+^] provides one example of translational readthrough modification in yeast cells. Previous works have shown that [PSI^+^] can reveal hidden phenotypic variation among yeast strains, that this effect is largely recapitulated by the *sup35^C653R^* mutation, which increases translational readthrough, and that the resulting phenotypic differences may have a complex genetic basis [9], [16]. Here, we have advanced our understanding of the genetic basis of readthrough-dependent phenotypes by identifying specific loci that underlie hidden variation revealed by *sup35^C653R^*. Using this partial-loss-of function allele of *SUP35* allowed us to focus on distinct hidden phenotypes in BY and RM revealed by increased translational readthrough.

Our growth rate measurements in diverse stressful conditions for wildtype and *sup35* BY and RM showed that *sup35*-mediated differences in growth between these two strains were relatively modest compared to previous studies of [PSI^+^]-mediated effects [9], [16]. This could potentially be explained by other [PSI^+^]-dependent phenotypic effects in yeast, such as ribosomal frame shifting [10] or the presence of Sup35 prion aggregates [20], which are absent in our system. We consider it more likely, given the reported recapitulation of most [PSI^+^] strain-dependent phenotypic effects with the *sup35^C653R^* mutation [9], that this difference in effect sizes is due to the different genetic backgrounds used.

We found that *sup35*-mediated increase in readthrough had different effects on growth rates in BY and RM for about one-quarter of the growth conditions tested. Our mapping results lend additional support to the previously reported inference that some readthrough-dependent growth phenotypes are genetically complex based on their segregation patterns [9], and further suggest that some of the underlying loci have small effect sizes.

We showed that *SKY1* is the causal gene underlying the strongest readthrough-dependent locus detected for growth in the presence of diamide and hydrogen peroxide. Our results suggest that translation of the BY downstream sequence of *SKY1* is disadvantageous for growth in these conditions. We found that a complex interplay between the genetic background, *SKY1* allelic state, and *sup35* determines growth rate in these two conditions. *SKY1* mRNA measurements showed that the readthrough-dependent effect of *SKY1* on the growth differences between BY and RM is not mediated by mRNA levels. However, we observed a dramatic drop in the *SKY1* mRNA level in *sup35* RM relative to wildtype RM, while we did not see a drop in the *SKY1* mRNA level in *sup35* BY relative to wildtype BY. One mechanism that could explain the *sup35*-mediated drop in the *SKY1* mRNA level in RM is Nonstop mRNA Decay (NSD) pathway [21], which might be differentially active in BY and RM. This mRNA surveillance mechanism is initiated when the ribosome reaches the 3′ end of the mRNA, and therefore eliminates transcripts lacking stop codons or transcripts that have stop codons that were bypassed during translation. Ribosomes are more likely to reach the 3′ end of an mRNA after reading through one or more stop codons in *sup35* strains than in wildtype strains. Therefore, NSD is also more likely to eliminate such mRNAs in the presence of *sup35*. Importantly, even a single ribosome that reaches the 3′ end of an mRNA is predicted to trigger NSD, resulting in the reduction of the mRNA level [22]. The decrease in *SKY1* mRNA caused by *sup35* in the RM background is largely rescued by swapping in the BY allele of *SKY1*, which suggests that the combination of increased readthrough and NSD can act in an allele-specific fashion.

Sky1p is a protein kinase that phosphorylates SR proteins [23], proteins with domains containing alternating serine and arginine residues which are components of the machinery for the processing [24] and nuclear export [25] of mRNAs. One of the known Sky1p targets, Npl3p, was shown to promote translation termination accuracy in yeast [26]. However, the same paper showed that the role of Npl3p in translation termination is independent of the posttranslational modifications mediated by Sky1p. Sky1p has also been shown to regulate cation homeostasis and salt tolerance [27]. Deletion of *SKY1* confers resistance to several anticancer drugs, such as cisplatin and carboplatin [28], and to polyamine toxic analogues [29]. Several hypotheses have been proposed to explain the role of Sky1p in resistance to these drugs, such as a Sky1p-mediated effect on splicing or transport of target mRNAs, regulation of membrane permeability, and regulation of drug uptake. However, the direct target(s) of Sky1p that mediate these effects are unknown. Our results demonstrate that deletion of *SKY1* also confers a growth advantage in the presence of oxidative stress inducers diamide and hydrogen peroxide. A genetic interaction between *SKY1* and *SUP35* was previously reported in the S288c background in rich media [30]. Here we showed that a genetic interaction is present between *SKY1* and *SUP35* in the presence of diamide and hydrogen peroxide in the BY background but not in the RM background. These results support previous finding that readthrough-dependent phenotypes vary based on the genetic background [9].

## Materials and Methods

### Strains, media, and plasmids

Cultures were grown in minimal medium containing 0.67% (w/v) yeast nitrogen base without amino acids (Difco) containing 2% (w/v) glucose (SMD) or rich medium, as specified. Additional nutritional supplements or drugs were added as required. YPD plates were made as described [31]. For sporulation, SPO++ was used (http://www.genomics.princeton.edu/dunham/sporulationdissection.htm).

We used pEF675 (a kind gift from Eric Foss) to replace *SUP35* with *sup35^C653R^* via two-step allele replacement [32] in BY4724 (*Mat* ***a*** *ura3Δ lys2Δ*) [33] and RM11-1a (*Mat* ***a*** *ura3Δ his3Δ0::NatMX hoΔ::HphMX AMN1^BY^*). pEF675 was made by sub-cloning *Sup35* into a common URA3-marked integrating yeast plasmid (pRS306 [34]) and subsequently changing cysteine 563 (TGT) to an arginine (CGT). Successful replacement for each strain was then confirmed by sequencing. We refer to strains with *SUP35* as wildtype and strains with *sup35^C653R^* as *sup35*. We then transferred *sup35* into strains with suitable genetic markers for X-QTL. To do so, we crossed BY-*sup35* and RM-*sup35* strains into BY MAT**α** *can1Δ::STE2pr-SpHIS5 lyp1Δ his3Δ1* and RM MAT**α** *AMN1^BY^ his3Δ0::NatMX hoΔ::HphMX* [14], respectively. After sporulating the obtained diploids and genotyping the dissected tetrads, we selected BY Mat**α** *his3Δ1 lyp1Δ can1Δ::STE2prSpHIS5 sup35^C653R^* and RM Mat**a** *AMN1^BY^ his3Δ0::NATMX hoΔ::HphMX sup35^C653R^* as *sup35* parental strains in X-QTL. We used strains form [14] as wildtype parental strains in X-QTL.

*SKY1* and *MRE11* replacement strains were generated by a two-step replacement method [35]. Each gene was first replaced with *URA3-KanMX* cassette from pCORE in wildtype and *sup35* BY and RM strains generating *goi*Δ::*URA3-KanMX* knockout strains. *SKY1* and *MRE11* alleles from the donor strains were amplified by PCR and introduced into recipient strains to replace *URA3-KanMX* cassette. Where mentioned, 400-base pair from the upstream regulatory region or 300-base pair from the downstream region is included in making replacement strains. Allele replacements were confirmed by sequencing.

### Sequencing

The RM *SKY1* and *MRE11* sequences were obtained from the whole genome-sequencing project at the Broad Institute (http://www.broad.mit.edu/annotation/genome/saccharomyces_cerevisiae/Home.html).

All sequencing was done using standard dideoxy methods.

### Growth rate measurement

We inoculated strains under examination in a 96-well plate (Costar; 3370) in rich medium and incubated the plate in 30°C until saturation. We then used a sterile 96-pin replicator (Nunc; 62409-606) to inoculate Costar 96-well plate (3370) containing rich medium and the reagent of interest. We then used Synergy 2 Multi-Mode Microplate Reader (BioTek Instruments) set at the desired temperature (30°C unless mentioned otherwise) and with continuous shaking at medium speed to collect OD_600_ at 30-minute intervals for up to 20 hours. We used data points corresponding to 0.05<OD_600_<0.5 (logarithmic growth phase) to calculate growth rate. Growth rate was calculated as the slope of a linear regression of the log transformed logarithmic growth phase data points. For each strain, unless otherwise specified, growth rate in rich media plus the reagent of interest is normalized by the strain's growth rate in rich media. Growth rate is shown as the mean ± the standard deviation of values obtained from at least eight independent growth measurements, including at least four biological replicates. We then performed t-test comparison between BY and RM growth rate ratios for all growth conditions. We used the obtained *p*-values to estimate the FDR at *p*-value<0.05 based on *q*-value calculation [36] in R (http://www.r-project.org/).

### Dual luciferase assay

Dual luciferase assay was performed as explained before [12]. Plasmids with the stop codon (pDB691) or the sense codon (pDB690), kindly provided by David Bedwell (University of Alabama at Birmingham), were transformed into the indicated yeast strains, and transformants were selected on SMD drop-out plates lacking uracil. Transformed strains were grown in liquid SMD medium to a cell density of 0.5–0.7 A600 units/mL as measured using Synergy 2 Multi-Mode Microplate Reader (BioTek Instruments). The luciferase assay was performed using the Dual-Luciferase Reporter Assay System (Promega; E1910). Approximately 10^4^ yeast cells from each strain expressing the indicated dual luciferase reporter were lysed using 100 µL of Passive Lysis Buffer in a 96-well plate (Costar; 3370). Two microliters of the lysate were added to 10 µL of the Luciferase Assay Reagent II in an opaque 96-well plate (Costar; 3614). Relative luminescence units (RLUs) produced by firefly luciferase activity were then measured for 10 seconds using Synergy 2 Multi-Mode Microplate Reader (BioTek Instruments). 10 µL of Stop&Glo buffer was then added to quench the firefly activity and activate the *Renilla* luciferase activity. RLUs were again measured for 10 seconds to determine the *Renilla* luciferase activity. Negative controls that contained all the reaction components except cell lysates were used to determine the background for each luciferase reaction and were subtracted from the experimental values obtained. Percent readthrough is expressed as the mean ± the standard deviation of values obtained from at least eight independent dual luciferase assay including at least four biological replicates.

### X-QTL

For each growth condition, we performed X-QTL on two biological replicates for the wildtype BY×RM cross and two biological replicates for the *sup35* cross. MAT**a** haploid segregants from the indicated cross were selected as explained before [14]. To create the segregating pool, a single colony of the diploid progenitor of the mentioned cross was inoculated into 5 mL YPD and grown to stationary phase. The diploid culture was spun down and the supernatant was decanted. The diploid pellet was then resuspended in 50 mL SPO++ sporulation medium. The sporulation was kept at room temperature (∼22°C) with shaking and monitored for the fraction of diploids that had sporulated. Once more than 50% of the diploids had sporulated, 10 mL of the sporulation were spun down and then the supernatant was decanted. The pellet was resuspended in 2 mL water. 600 µL β-glucoronidase (Sigma; G7770) were added to the preparation, and the mixture was incubated at 30°C for one hour. Water was added to the sample to the total volume of 20 mL. The spore preparation was spread onto SMD+canavanine/thialysine plates (Sigma; C9758 for canavanine (L-canavanine sulphate salt); A2636 for thialysine (S-(2-aminoethyl)- L-cysteine hydrochloride)), with 100 µL of sample going onto each plate. The plates were incubated at 30°C for two days. Then 10 mL of water were poured onto each plate and a sterile spreader was used to remove the segregants from the plate. The cell mixtures from four plates were then pipetted off the plates into a container. The pool was spun down and the water decanted. Haploid segregants were then inoculated into 100 mL liquid YPD and were incubated in 30°C while shaking on a rotary shaker at 200 rpm for about 30 minutes to recover. After the recovery, 100 µL of the segregant pool was pipetted and spread on the selection plates (YPD+reagent of interest) and control plates (YPD). For each condition/cross we used five selection plates to pool the resistant segregants. For each cross, we used three control plates to pool the whole population of segregants. Selection and control plates were then incubated at 30°C for two days. DNA was extracted from the grown cells using Genomic-tip 100/G columns (Qiagen; 10243). DNA was labeled using the BioPrime Array CGH Genomic Labeling Module (Invitrogen; 18095-012) with the sample being labeled with Cy3 dUTP and the reference being labeled with Cy5 dUTP. We used a BY/RM diploid as the reference for all hybridizations. Labeled samples were then hybridized onto the allele-specific genotyping microarray with isothermal probes that assay ∼18,000 single nucleotide polymorphisms (SNPs) between BY and RM [14]. The array data have been deposited in NCBI's Gene Expression Omnibus (GEO) [37] and are accessible through GEO Series accession number GSE33817 (http://www.ncbi.nlm.nih.gov/geo/query/acc.cgi?acc=GSE33817). Hybridization intensities were extracted and normalized using the rank invariant method in the Agilent Feature Extraction software package. For a given SNP, the difference in the log_10_ ratios of BY and RM-specific probes on a single array (or log_10_ intensity difference) was computed. Background allele frequency changes that occur during pool construction were removed from the selection by subtracting the log_10_ intensity difference obtained for the whole (control) population from the log_10_ intensity difference observed in the selections. To find readthrough-dependent peaks, we used a Savitzky-Golay filter to smooth the input data within sliding windows of 100 probes. The Savitzky-Golay method essentially performs a local polynomial regression on a series of values to determine the smoothed value for each point. This smoothing approach was used to preserve local maxima in the data. For each probe, we subtracted the average of the two wildtype X-QTL replicates from the average of the two sup35 X-QTL replicates for each growth condition and used this measure as the input data. Readthrough-dependent loci were called if the smoothed value surpassed the threshold for a 5% FDR, where the number of false discoveries at each threshold was determined by using the same algorithm on the control data, which were obtained by subtracting wildtype control X-QTL results from sup35 control X-QTL results for growth on YPD. To ensure that the results are robust to the statistical approach used, we also performed a student's t-test comparison of a moving window of six probes from the background-subtracted data for the two wildtype and the two mutant replicates for each selection. We set the threshold at *p value*<2.78×10^−6^ (Bonferroni-corrected *p*<0.05). Peak calling and all other statistical analyses were conducted in R (http://www.r-project.org/).

### ANOVA

For growth rate in diamide and hydrogen peroxide, an ANOVA of the form
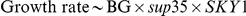
was performed in R using the *lm* function. BG stands for genetic background, which can be either BY or RM. *sup35* stands for the allelic status of *SUP35*, which can be either wildtype or *sup35^C653R^*. *SKY1* stands for the allelic status of *SKY1*, which can be either BY allele or RM allele. To test whether *SKY1* mRNA level could account for *SKY1* allelic effect, we first used an ANOVA of the form
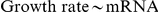
where mRNA stand for *SKY1* mRNA level and then used the residuals in an ANOVA of the form mentioned above.

### Quantitative RT–PCR

Each quantitative RT-PCR measurement represents data collected from three biological replicates. We harvested cells from the logarithmically growing strains in the mentioned growth conditions. We then used total RNA extraction kit (Norgen; 17200) to extract total RNA from collected cells. To perform quantitative RT-PCR, we used TaqMan RNA-to-C_T_ one-step kit (Applied Biosystems; 4393463C) and 7900HT Fast Real-Time PCR System (Applied Biosystems; 4329001). We used TaqMan TAMRA probes. We used a 6-FAM labeled probe for *SKY1* detection and a VIC labeled probe for *TDH2* detection (internal control). The primers were selected so that there would be no polymorphisms in the sequence amplified for *SKY1* (185-base pair fragment starting at 403^rd^ nucleotide of *SKY1* coding sequence) and *TDH2* (168-base pair fragment starting at 379^th^ nucleotide of *TDH2* coding sequence). The sequences of primers and probes used are as follows:

*SKY1*-F: ATGTGACGAAAGGAACGAAGA

*SKY1*-R: ACTAAAATGTAGCGTGCATCCTT

*SKY1*-probe: TCTTTGAAAGATTACAGGCCGGGTG

*TDH2*-F: AGGTTGTCATCACTGCTCCAT

*TDH2*-R: GTGGTACAAGAAGCGTTGGAA

*TDH2*-probe: CCAATGTTCGTCATGGGTGTTAACG

## Supporting Information

Figure S1 Partial loss of function allele of *SUP35* (*sup35^C653R^*) increases readthrough in BY and RM. Replacing wildtype allele of *SUP35* with the partial loss of function allele (*sup35^C653R^*) increases %readthrough in both BY and RM. %Readthrough was measured via a dual luciferase reporter assay, which uses tandem *Renilla* and firefly luciferase genes that are separated by a single in-frame stop codon. The activity of the firefly luciferase, encoded by the distal open reading frame, provides a quantitative measure of the readthrough of the stop codon that separates the two open reading frames. The activity of the *Renilla* luciferase, encoded by the proximal open reading frame, serves as an internal control for mRNA abundance. Thus, the relative abundance of these light-emitting proteins measures the efficiency of translation termination. Here, we used two separate reporters; one with UGA (stop codon) and one with CGA (sense codon) separating the *Renilla* and firefly open reading frames. For each strain, we calculated the readthrough as the ratio of firefly to Renilla luciferase activity in the presence of the stop codon, normalized by the observed ratio for the sense codon constructs.
(TIF)

Figure S2 A. X-QTL results for growth on chlorpromazine. Result for segregants from a cross between *sup35* BY and RM, wildtype BY and RM, and the t-test comparison between wildtype and *sup35* results is shown. The top two plots show comparisons of the allele frequencies from selected segregating population (Selection) to the whole population (Control) from a cross between *sup35* parent strains (*sup35*) and from a cross between wildtype parent strains (WT). For plotting, average of two biological replicates is used for each selection and average of six biological replicates is used for each control. Sliding window averages (40 kb) are plotted. Enrichment of the BY allele is indicated by deviations above zero and enrichment of the RM allele is indicated by deviations below zero. The third plot shows the readthrough-dependent loci (marked with dotted lines) called using an smoothing algorithm based on Savitzky-Golay filter on the differences between allele-frequency skews for wildtype and *sup35* X-QTL results (FDR 5%, Materials and Methods). The bottom plot shows −Log_10_(*p*) obtained from t-test comparison between allele frequencies in wildtype and *sup35* selected pools. When present, the readthrough-dependent loci (*p*<2.78×10^−6^; Bonferroni-corrected *p*<0.05) are marked with dotted lines. Results are represented in the same manner for (B–I). B. X-QTL results for growth on cobalt chloride. C. X-QTL results for growth on cycloheximide. D. X-QTL results for growth on diamide. E. X-QTL results for growth on E6-berbamine. F. X-QTL results for growth on ethanol. G. X-QTL results for growth on hydrogen peroxide. H. X-QTL results for growth on neomycin. I. X-QTL results for growth on tunicamycin.
(TIFF)

Figure S3 Interval corresponding to readthrough-dependent locus detected for growth in presence of diamide on Chromosome XIII. 50 kb surrounding the region on chromosome XIII and the genes residing in the region is shown (http://www.yeastgenome.org/).
(TIF)

Figure S4 *SKY1* variation underlies the locus on Chromosome XIII for growth on hydrogen peroxide. A) Comparisons of the selected segregating population (Selection) to the whole population (Control) from a cross between *sup35* BY and RM parent strains (*sup35*) and a cross between wildtype parent strains (WT) are shown for *MRE11*-fixed populations (BY×RM- *MRE11* ^BY^) in hydrogen peroxide. For plotting, average of two biological replicates is used for each selection and control. B) Comparisons of the selected segregating population (Selection) to the whole population (Control) from a cross between *sup35* BY and RM parent strains (*sup35*) and a cross between wildtype parent strains (WT) are shown for *SKY1*-fixed populations (BY×RM- *SKY1* ^BY^) in hydrogen peroxide. For plotting, average of two biological replicates is used for each selection and control. The dotted line shows the interval surrounding *MRE11* and *SKY1*.
(TIF)

Figure S5 *SKY1* variation contributes to readthrough-dependent differences in growth rates of BY and RM on H_2_O_2_. A) Knocking out *SKY1* eliminates the observed difference between wildtype and *sup35* growth rates in BY background. B) Replacing *SKY1* (upstream regulatory region, open reading frame and the downstream sequence) in the BY background with the RM allele increased growth rate in diamide in the presence of *sup35*, and replacing *SKY1* (upstream regulatory region, open reading frame and the downstream sequence) in the RM background with the BY allele decreased growth rate in the presence of *sup35*. C and D) these panels show the data presented in panels A and B grouped in different ways to highlight the growth effects of the *sup35* allelic state and the growth effects of the genetic background. Growth rates of wildtype and *sup35* BY and RM, as well as the corresponding *SKY1* swapped and *sky1Δ* strains are shown for growth in the presence of hydrogen peroxide, grouped according to the *sup35* allelic state (C) and genetic background (D). For each strain, growth rates are normalized based on the strain's growth rate in rich medium (YPD).
(TIF)

Figure S6 Readthrough-dependent strain-specific growth effects of *SKY1* in hydrogen peroxide. Replacing the *SKY1* downstream sequence alone captures the allelic effects of *SKY1* in *sup35* strains. Introducing a second stop codon immediately after the native stop codon at the end of the *SKY1* open reading frame shows that strain-specific growth effects of *SKY1* polymorphism are readthrough-dependent.
(TIF)

Table S1 List of growth conditions tested.
(DOC)

Table S2 Modeling growth rate in the presence of hydrogen peroxide using ANOVA with three factors: genetic background (BG), *SUP35* allelic status, and *SKY1* allelic status.
(DOC)

Table S3 Modeling growth rate in the presence of hydrogen peroxide after regressing out *SKY1* mRNA data using ANOVA with three factors: genetic background (BG), *SUP35* allelic status, and *SKY1* allelic status.
(DOC)
