# Supplementary figures and images for: Genetic Basis of Hidden Phenotypic Variation Revealed by Increased Translational Readthrough in Yeast
Source: PLoS Genet. 2012 Mar 1;8(3):e1002546. doi: 10.1371/journal.pgen.1002546 (PMC3291563; doi:10.1371/journal.pgen.1002546)

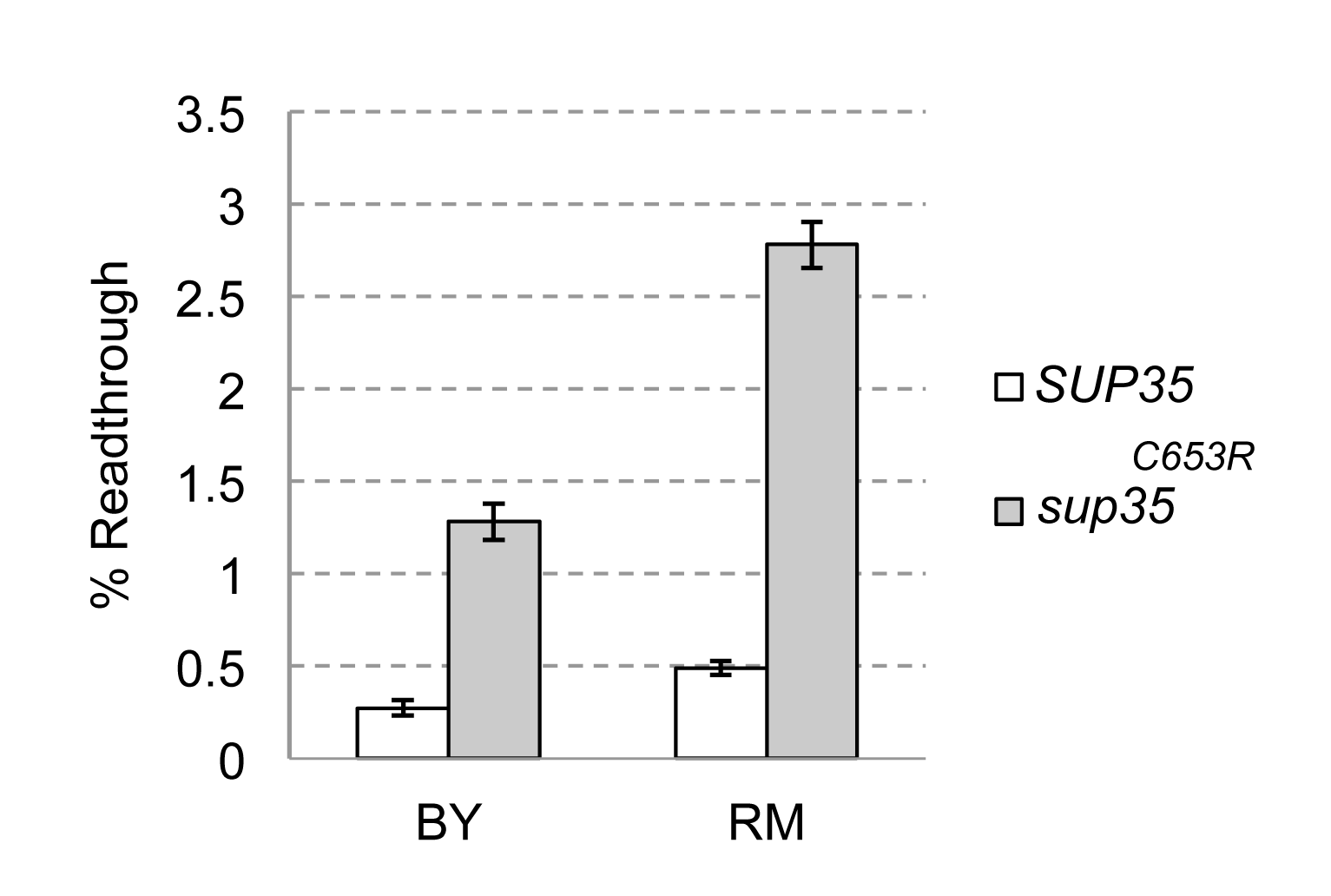

Supplement: Figure S1 — Partial loss of function allele of SUP35 (sup35C653R) increases readthrough in BY and RM. Replacing wildtype allele of SUP35 with the partial loss of function allele (sup35C653R) increases %readthrough in both BY and RM. %Readthrough was measured via a dual luciferase reporter assay, which uses tandem Renilla and firefly luciferase genes that are separated by a single in-frame stop codon. The activity of the firefly luciferase, encoded by the distal open reading frame, provides a quantitative measure of the readthrough of the stop codon that separates the two open reading frames. The activity of the Renilla luciferase, encoded by the proximal open reading frame, serves as an internal control for mRNA abundance. Thus, the relative abundance of these light-emitting proteins measures the efficiency of translation termination. Here, we used two separate reporters; one with UGA (stop codon) and one with CGA (sense codon) separating the Renilla and firefly open reading frames. For each strain, we calculated the readthrough as the ratio of firefly to Renilla luciferase activity in the presence of the stop codon, normalized by the observed ratio for the sense codon constructs. (TIF) [file pgen.1002546.s001.tif]

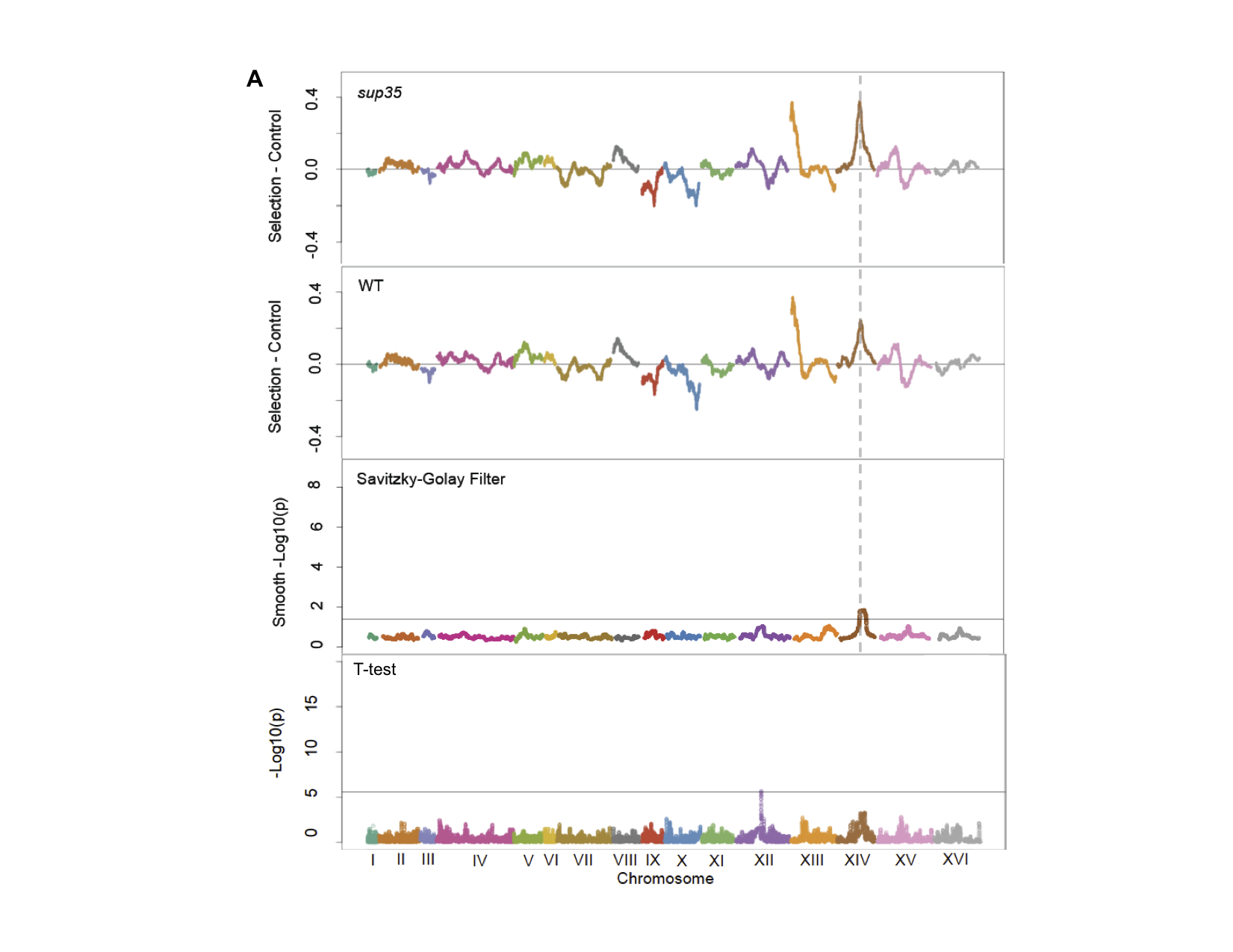

Supplement: Figure S2 — A. X-QTL results for growth on chlorpromazine. Result for segregants from a cross between sup35 BY and RM, wildtype BY and RM, and the t-test comparison between wildtype and sup35 results is shown. The top two plots show comparisons of the allele frequencies from selected segregating population (Selection) to the whole population (Control) from a cross between sup35 parent strains (sup35) and from a cross between wildtype parent strains (WT). For plotting, average of two biological replicates is used for each selection and average of six biological replicates is used for each control. Sliding window averages (40 kb) are plotted. Enrichment of the BY allele is indicated by deviations above zero and enrichment of the RM allele is indicated by deviations below zero. The third plot shows the readthrough-dependent loci (marked with dotted lines) called using an smoothing algorithm based on Savitzky-Golay filter on the differences between allele-frequency skews for wildtype and sup35 X-QTL results (FDR 5%, Materials and Methods). The bottom plot shows −Log10(p) obtained from t-test comparison between allele frequencies in wildtype and sup35 selected pools. When present, the readthrough-dependent loci (p<2.78×10^−6^; Bonferroni-corrected p<0.05) are marked with dotted lines. Results are represented in the same manner for (B–I). B. X-QTL results for growth on cobalt chloride. C. X-QTL results for growth on cycloheximide. D. X-QTL results for growth on diamide. E. X-QTL results for growth on E6-berbamine. F. X-QTL results for growth on ethanol. G. X-QTL results for growth on hydrogen peroxide. H. X-QTL results for growth on neomycin. I. X-QTL results for growth on tunicamycin. (TIFF) [file pgen.1002546.s002.tiff]

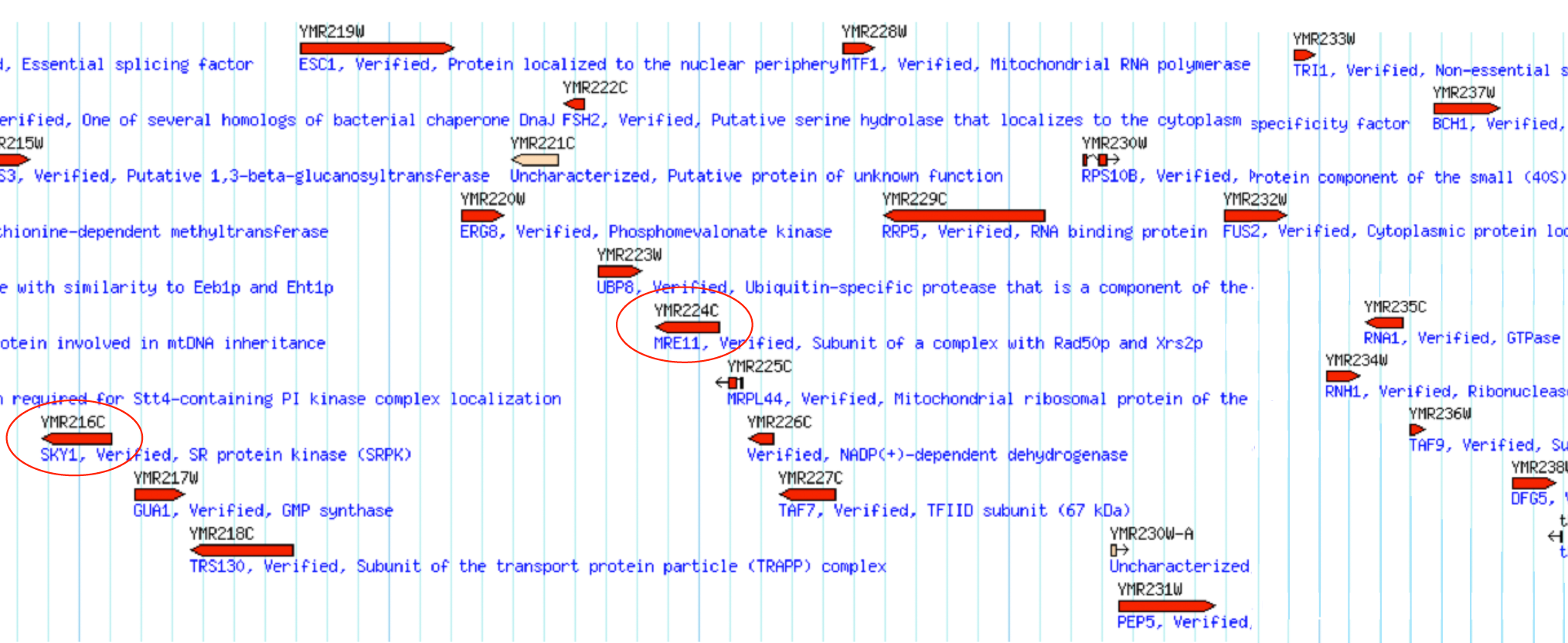

Supplement: Figure S3 — Interval corresponding to readthrough-dependent locus detected for growth in presence of diamide on Chromosome XIII. 50 kb surrounding the region on chromosome XIII and the genes residing in the region is shown (http://www.yeastgenome.org/). (TIF) [file pgen.1002546.s003.tif]

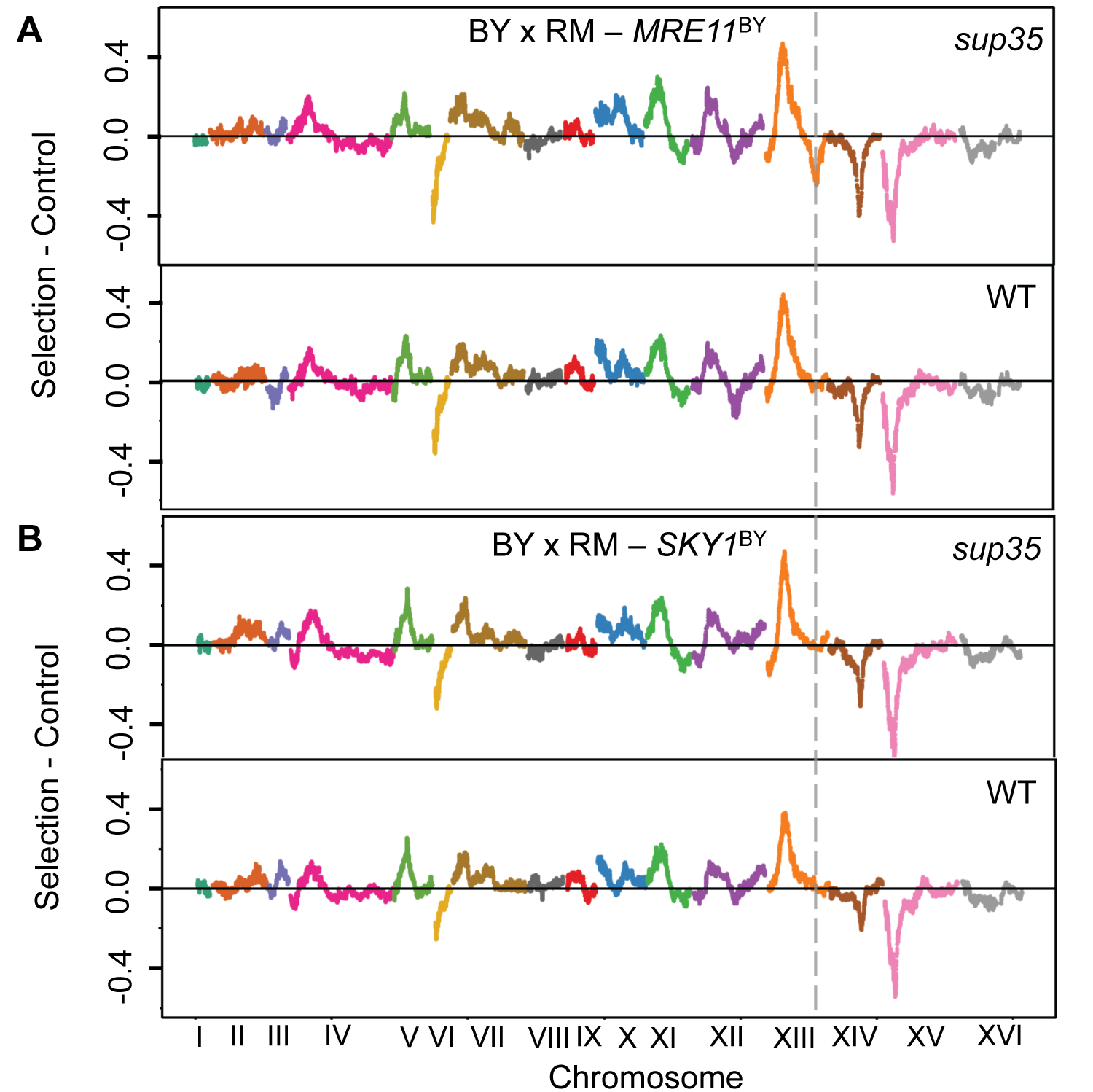

Supplement: Figure S4 — SKY1 variation underlies the locus on Chromosome XIII for growth on hydrogen peroxide. A) Comparisons of the selected segregating population (Selection) to the whole population (Control) from a cross between sup35 BY and RM parent strains (sup35) and a cross between wildtype parent strains (WT) are shown for MRE11-fixed populations (BY×RM- MRE11 BY) in hydrogen peroxide. For plotting, average of two biological replicates is used for each selection and control. B) Comparisons of the selected segregating population (Selection) to the whole population (Control) from a cross between sup35 BY and RM parent strains (sup35) and a cross between wildtype parent strains (WT) are shown for SKY1-fixed populations (BY×RM- SKY1 BY) in hydrogen peroxide. For plotting, average of two biological replicates is used for each selection and control. The dotted line shows the interval surrounding MRE11 and SKY1. (TIF) [file pgen.1002546.s004.tif]

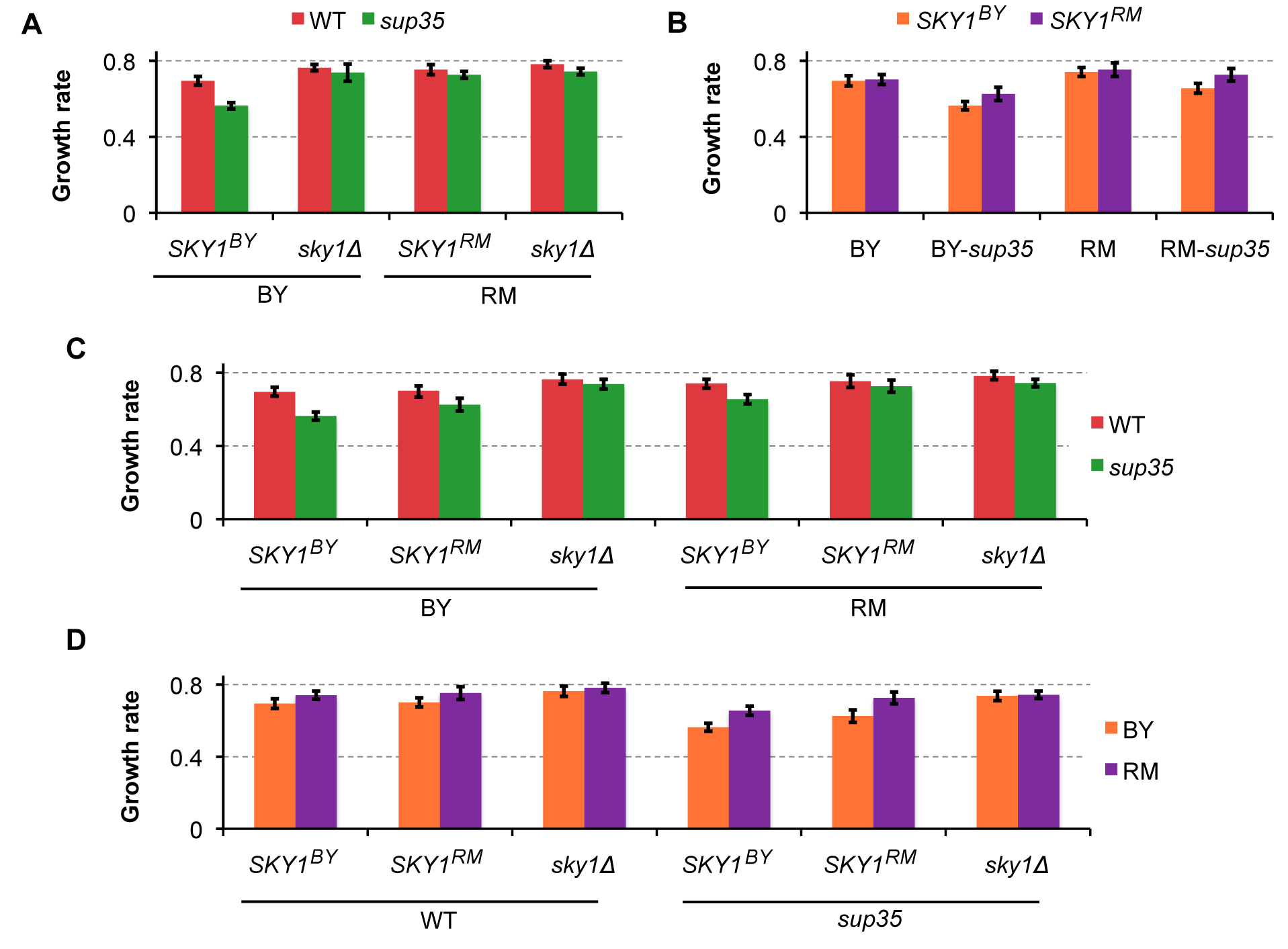

Supplement: Figure S5 — SKY1 variation contributes to readthrough-dependent differences in growth rates of BY and RM on H2O2. A) Knocking out SKY1 eliminates the observed difference between wildtype and sup35 growth rates in BY background. B) Replacing SKY1 (upstream regulatory region, open reading frame and the downstream sequence) in the BY background with the RM allele increased growth rate in diamide in the presence of sup35, and replacing SKY1 (upstream regulatory region, open reading frame and the downstream sequence) in the RM background with the BY allele decreased growth rate in the presence of sup35. C and D) these panels show the data presented in panels A and B grouped in different ways to highlight the growth effects of the sup35 allelic state and the growth effects of the genetic background. Growth rates of wildtype and sup35 BY and RM, as well as the corresponding SKY1 swapped and sky1Δ strains are shown for growth in the presence of hydrogen peroxide, grouped according to the sup35 allelic state (C) and genetic background (D). For each strain, growth rates are normalized based on the strain's growth rate in rich medium (YPD). (TIF) [file pgen.1002546.s005.tif]

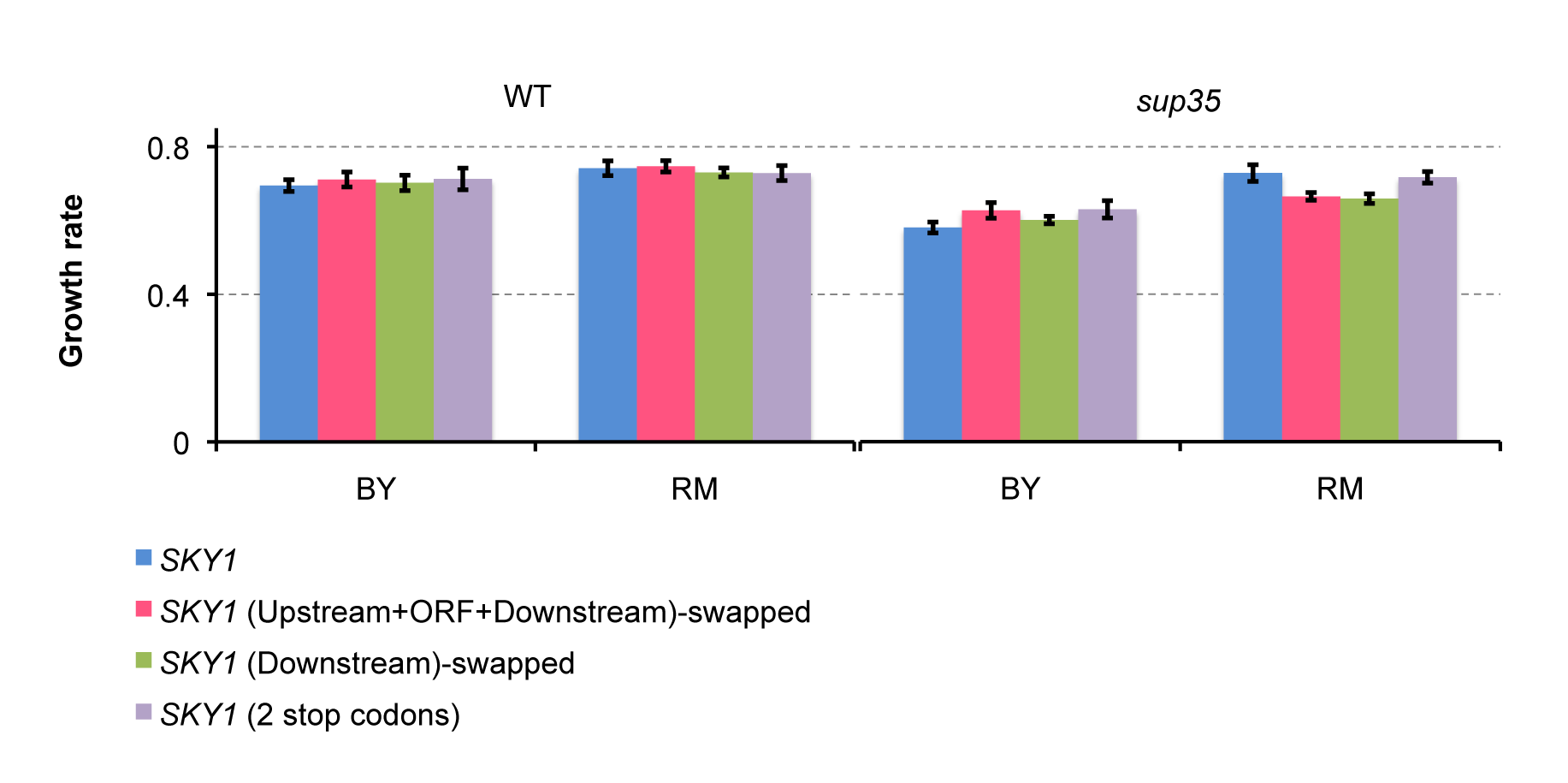

Supplement: Figure S6 — Readthrough-dependent strain-specific growth effects of SKY1 in hydrogen peroxide. Replacing the SKY1 downstream sequence alone captures the allelic effects of SKY1 in sup35 strains. Introducing a second stop codon immediately after the native stop codon at the end of the SKY1 open reading frame shows that strain-specific growth effects of SKY1 polymorphism are readthrough-dependent. (TIF) [file pgen.1002546.s006.tif]
