# Supplementary material for: Genetic Basis of Hidden Phenotypic Variation Revealed by Increased Translational Readthrough in Yeast
Source: PLoS Genet. 2012 Mar 1;8(3):e1002546. doi: 10.1371/journal.pgen.1002546 (PMC3291563; doi:10.1371/journal.pgen.1002546)
Supplement: Table S1 — List of growth conditions tested. (DOC) [file pgen.1002546.s007.doc]

Table S1. List of growth conditions tested.

| Growth Condition | Dose-response range | Figure 1 Concentration | X-QTL Concentration | Unit | Source |
| --- | --- | --- | --- | --- | --- |
| Chlorpromazine | 2 - 10 | 8 | 20 | mM | Fluka |
| CoCl_2_ | 0.25 - 1.5 | 1 | 3 | M | Sigma |
| Cycloheximide | 5 - 30 | 25 | 100 | ng/mL | Sigma |
| Diamide | 0.25 - 1.5 | 1 | 2 | mM | Sigma |
| E6-berbamine | 2 - 10 | 8 | 40 | mM | Enzo Life Sciences |
| Ethanol | 2 - 10% | 6% | 8% | V/V | Sigma |
| H_2_O_2_ | 2 - 10 | 7 | 36 | mM | Fisher |
| Neomycin | 1 - 7 | 5 | 10 | mg/mL | Sigma |
| Tunicamycin | 0.1 - .075 | 0.6 | 3 | mM | Biomol |
| Anisomycin | 20 - 50 | NA | NA | μM | Biomol |
| Benomyl | 2 - 30 | NA | NA | mg/mL | Chem Service |
| Caffeine | 1 - 15 | NA | NA | mM | Sigma |
| FCCP | 5 - 40 | NA | NA | μM | Sigma |
| Menadione | 15 - 60 | NA | NA | μM | Sigma |
| Penitrum A | 10 - 40 | NA | NA | μM | Enzo Life Sciences |
| Mastoparan | 5 - 20 | NA | NA | μM | Calbiochem |
| Paraquat | 0.5 - 3 | NA | NA | mM | ChemService |
| Doxorubicin | 10 - 40 | NA | NA | μM | Biomol |
| LY83583 | 20 - 100 | NA | NA | μM | Biomol |
| Manumycin | 5 - 30 | NA | NA | μM | Biomol |
| Rapamycin | 25 - 200 | NA | NA | nM | Sigma |
| Nystatin | 2 - 40 | NA | NA | μg/mL | Sigma |
| NaCl | 0.5 - 2 | NA | NA | M | Sigma |
| KCl | 0.5 - 2 | NA | NA | M | Sigma |
| AmOAc | 0.25 - 1 | NA | NA | M | Sigma |
| CdCl_2_ | 0.1 - 2 | NA | NA | M | Sigma |
| Temperature | 20°C | NA | NA | NA | NA |
| Temperature | 28°C | NA | NA | NA | NA |
| Temperature | 33°C | NA | NA | NA | NA |
| Temperature | 36°C | NA | NA | NA | NA |
| Carbon Source Galactose | 2% | NA | NA | W/V | Sigma |
| Carbon Source Ethanol | 1% | NA | NA | V/V | Sigma |
