# Supplementary material for: Genetic Basis of Hidden Phenotypic Variation Revealed by Increased Translational Readthrough in Yeast
Source: PLoS Genet. 2012 Mar 1;8(3):e1002546. doi: 10.1371/journal.pgen.1002546 (PMC3291563; doi:10.1371/journal.pgen.1002546)
Supplement: Table S2 — Modeling growth rate in the presence of hydrogen peroxide using ANOVA with three factors: genetic background (BG), SUP35 allelic status, and SKY1 allelic status. (DOC) [file pgen.1002546.s008.doc]

Table S2. Modeling growth rate in the presence of hydrogen peroxide using ANOVA with three factors: genetic background (BG), *SUP35* allelic status, and *SKY1* allelic status.

| **Coefficient** | **Estimate** | **Std. Error** | **t value** | **Pr(>|t|)** |
| --- | --- | --- | --- | --- |
| (Intercept) | 0.726 | 0.00933 | 77.81 | < 2e-16 |
| BG (BY) | -0.101 | 0.0132 | -7.63 | 3.15E-10 |
| SUP35 (WT) | 0.0272 | 0.0132 | 2.061 | 0.04393 |
| SKY1 (BY) | -0.0708 | 0.0132 | -5.363 | 1.61E-06 |
| BG (BY): SUP35 (WT) | 0.0487 | 0.0187 | 2.607 | 0.01168 |
| BG (BY): SKY1 (BY) | 0.00862 | 0.0187 | 0.462 | 0.64592 |
| SUP35 (BY): SKY1 (BY) | 0.0588 | 0.0187 | 3.152 | 0.00261 |
| BG (BY): SUP35 (WT): SKY1 (BY) | -0.00346 | 0.0264 | -0.131 | 0.89595 |
