# Supplementary material for: Genetic Basis of Hidden Phenotypic Variation Revealed by Increased Translational Readthrough in Yeast
Source: PLoS Genet. 2012 Mar 1;8(3):e1002546. doi: 10.1371/journal.pgen.1002546 (PMC3291563; doi:10.1371/journal.pgen.1002546)
Supplement: Table S3 — Modeling growth rate in the presence of hydrogen peroxide after regressing out SKY1 mRNA data using ANOVA with three factors: genetic background (BG), SUP35 allelic status, and SKY1 allelic status. (DOC) [file pgen.1002546.s009.doc]

Table S3. Modeling growth rate in the presence of hydrogen peroxide after regressing out *SKY1* mRNA data using ANOVA with three factors: genetic background (BG), *SUP35* allelic status, and *SKY1* allelic status.

| **Coefficient** | **Estimate** | **Std. Error** | **t value** | **Pr(>|t|)** |
| --- | --- | --- | --- | --- |
| (Intercept) | 0.0373 | 0.0095 | 3.927 | 0.000238 |
| BG (BY) | -0.0977 | 0.0134 | -7.274 | 1.22E-09 |
| SUP35 (WT) | 0.0297 | 0.0134 | 2.212 | 0.0311 |
| SKY1 (BY) | -0.0664 | 0.0134 | -4.943 | 7.33E-06 |
| BG (BY): SUP35 (WT) | 0.0458 | 0.0190 | 2.41 | 0.0193 |
| BG (BY): SKY1 (BY) | 0.0124 | 0.0190 | 0.65 | 0.518 |
| SUP35 (BY): SKY1 (BY) | 0.0673 | 0.0190 | 3.543 | 0.00081 |
| BG (BY): SUP35 (WT): SKY1 (BY) | -0.0117 | 0.0269 | -0.436 | 0.665 |
